# Study on safety of laparoscopic total gastrectomy for clinical stage I gastric cancer: the protocol of the CLASS02–01 multicenter randomized controlled clinical trial

**DOI:** 10.1186/s12885-018-4846-z

**Published:** 2018-10-03

**Authors:** Hongyong He, Haojie Li, Xiangqian Su, Ziyu Li, Peiwu Yu, Hua Huang, Changming Huang, Jianxin Ye, Yong Li, Jian Suo, Jiren Yu, Guoxin Li, Zekuan Xu, Gang Zhao, Hui Cao, Jiankun Hu, Xiaohui Du, Fenglin Liu, Yihong Sun, on behalf of the Chinese Laparoscopic Gastrointestinal Surgery Study (CLASS) Group

**Affiliations:** 1 Department of General Surgery, Zhongshan Hospital, Fudan University, Fenglin Road 180, Shanghai, 200032 China; 2 Department of General Surgery, Beijing Cancer Hospital, Peking University, Beijing, 100142 China; 3 Department of General Surgery, Southwest Hospital, Third Military Medical University, Chongqing, 400038 China; 4 Department of General Surgery, Fudan University Shanghai Cancer Center, Shanghai, 200032 China; 5 Department of General Surgery, Fujian Medical University Union Hospital, Fuzhou, 350001 China; 6 Department of General Surgery, The First Affiliated Hospital of Fujian Medical University, Fuzhou, 350009 China; 7 Department of General Surgery, Guangdong General Hospital, Guangzhou, 510080 China; 8 Department of General Surgery, The First Bethune Hospital of Jilin University, Changchun, 130021 China; 9 Department of General Surgery, The First Affiliated Hospital, Zhejiang University, Hangzhou, 310003 China; 10 Department of General Surgery, Nangfang Hospital, Southern Medical University, Guangzhou, 510515 China; 11 Department of General Surgery, The First Affiliated Hospital With Nanjing Medical University, Nanjing, 210029 China; 12 Department of General Surgery, Renji Hospital, Shanghai Jiaotong University, Shanghai, 200240 China; 13 Department of General Surgery, West China Hospital, Sichuan University, Chengdu, 610041 China; 14 Department of General Surgery, Chinese PLA General Hospital, Beijing, 10853 China

**Keywords:** Gastric cancer, Laparoscopic total gastrectomy, Safety, Randomized controlled trial

## Abstract

**Background:**

The safety of laparoscopic total gastrectomy (LTG) for the treatment of gastric cancer remains lack of clinical evidence. The Chinese Laparoscopic Gastrointestinal Surgery Study (CLASS) Group recently launched a multicenter randomized clinical trial (CLASS02–01) to compare the safety of LTG for clinical stage I gastric cancer with the conventional open total gastrectomy (OTG).

**Methods:**

This CLASS02–01 trial is a prospective, multicenter, randomized, controlled, open, and non-inferiority trial. Two hundred patients who met the inclusion criteria and did not accord with the exclusion criteria will be randomly divided into LTG group (*n* = 100) and OTG group (n = 100). The primary purpose of this study is to evaluate the early operative morbidity and mortality of LTG compared with OTG for clinical stage I gastric adenocarcinoma. The second purpose is to evaluate the recovery course and compare the postoperative hospital stay of the patients enrolled in this study.

**Discussion:**

This CLASS02–01 trial is the first prospective randomized two-arm controlled study to determine the safety of LTG compared with OTG. Through this trial, we hope to show that experienced surgeons can safely perform LTG with lymphadenectomy for gastric cancer.

**Trial registration:**

ClinicalTrials.gov ID: NCT03007550. December 30, 2016.

## Background

Gastric cancer is still an important health problem nowadays, being the fourth most common cancer and the third leading cause of cancer-related death worldwide [1]. Age standardized mortality rates for gastric cancer are 14.3 per 100,000 in men and 6.9 per 100,000 in women [2]. Incidence shows clear regional and sex variations-rates are highest in Eastern Asia, Eastern Europe, and South America and lowest in Northern and Southern Africa [1]. More than 679,000 new cases and 498,000 deaths occur every year in China [2].

More than 20 years after the introduction of laparoscopic gastrectomy, many large-scale randomized controlled trials (RCT) have been conducted in Japan (JCOG0912 and JLSSG0901) [3, 4], Korea (KLASS01 and KLASS02) [5–9], and China (CLASS01) [10]. These trials are all designed to evaluate the non-inferiority of laparoscopic-assisted distal gastrectomy (LADG) to its open counterpart. No RCT for laparoscopic total gastrectomy (LTG) exists at this moment. At present, Japan (JCOG1401) [11] and Korea (KLASS03) [12] have planned or launched clinical studies on LTG. The standardization of techniques for esophagojejunal anastomosis in LTG has been difficult even for experienced surgeons [13]. China is one of the countries with the highest incidence of gastric cancer and surgeons have accumulated extensive experience through CLASS01 study. So, it’s time for conducting the clinical research on the safety of LTG for gastric cancer.

## Methods

### Objectives

**Fig. 1:**
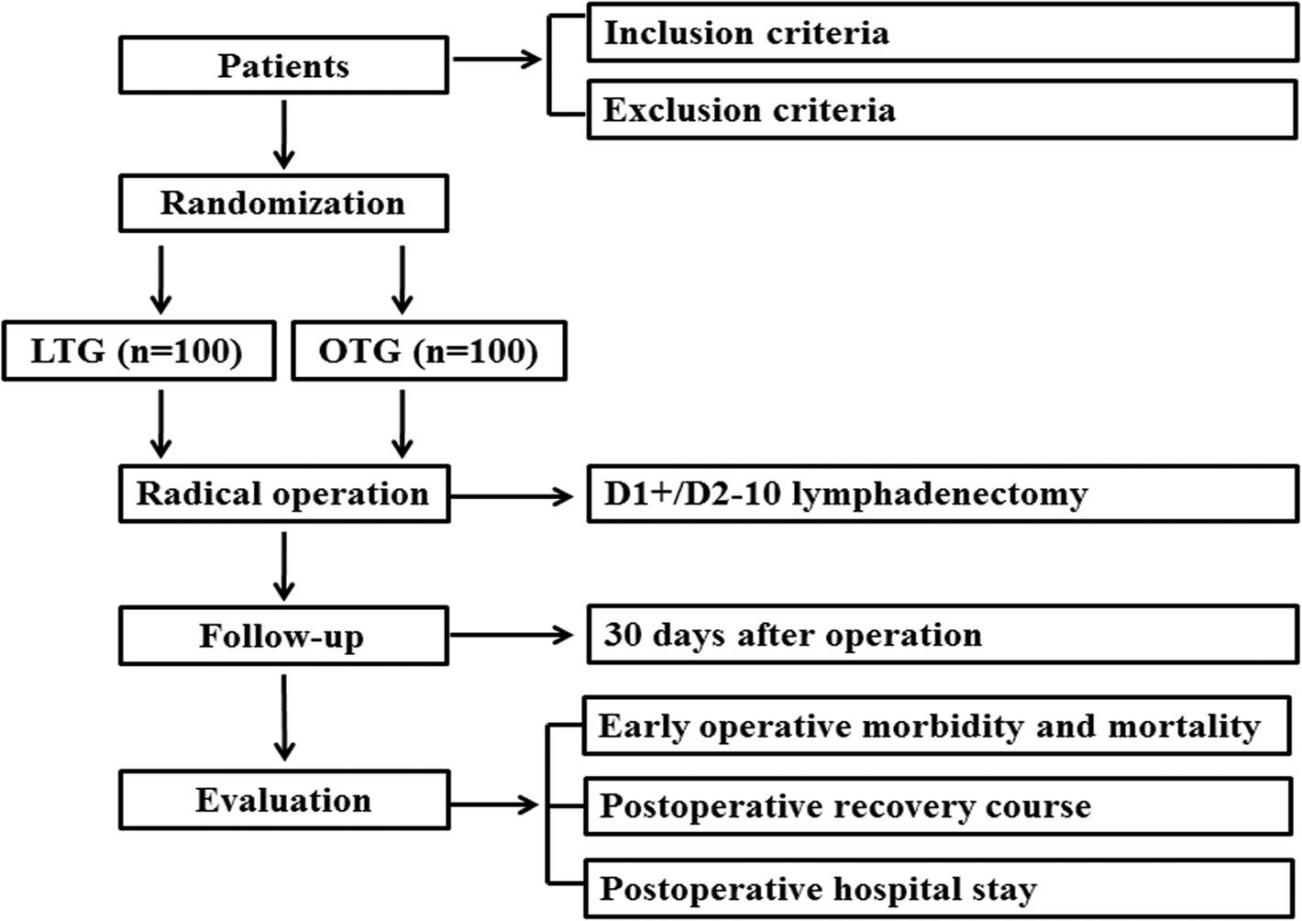
Study schema

This CLASS02–01 trial is a prospective, multicenter trial for laparoscopic total gastrectomy (LTG) and open total gastrectomy (OTG) in patients with clinical stage I (T1N0M0、T1N1M0、T2N0M0) gastric cancer. The primary purpose of this study is to evaluate the early operative morbidity and mortality and determine the safety of LTG compared with OTG for clinical stage I gastric adenocarcinoma. The second purpose is to evaluate the recovery course and compare the postoperative hospital stay of the patients enrolled in this study (Fig. 1).

### Study design

This CLASS02–01 trial is a prospective, multicenter, randomized, controlled, open, and non-inferiority trial comparing the safety of LTG (D1+/D2–10 lymphadenectomy) for clinical stage I (T1N0M0、T1N1M0、T2N0M0) gastric cancer in the upper and middle of their stomach with the conventional OTG.

### Ethic approval

Before enrollment of first patient, this study was approved from the institutional review boards of all research centers (14 tertiary hospitals) in China, including Zhongshan Hospital Fudan University (B2016-160R), Beijing Cancer Hospital, Southwest Hospital, Fudan University Shanghai Cancer Center, Fujian Medical University Union Hospital, The First Affiliated Hospital of Fujian Medical University, Guangdong General Hospital, The First Bethune Hospital of Jilin University, The First Affiliated Hospital Zhejiang University, Nangfang Hospital, The First Affiliated Hospital With Nanjing Medical University, Renji Hospital, West China Hospital, Sichuan University, and Chinese PLA General Hospital. Written informed consent will be obtained from all patients for the acquisition and use of anonymized clinical data before they are recruited, and all investigators will progress this study in accordance with the Declaration of Helsinki. This CLASS02–01 trial will be monitored by an independent data and safety monitoring committee (DSMC).

### Participating surgeons

To participate in this CLASS02–01 trial, the responsible surgeons should meet the following qualifications: first, completing at least 50 cases of OTG and LTG with D2 lymphadenectomy respectively; second, passing the blind review of surgery video. Briefly, the applicants should provide the videos of OTG and LTG in recent 3 months (three cases each) to the CLASS Research Council; CLASS Research Council will select two videos of OTG and LTG separately, and randomly appoint three experts to peer review blindly. When three experts unanimously approved it, the applicant will be permitted to participate in this study as a researcher.

### Sample size calculation

This is a non-inferiority verification study on clinical safety, with the early operative morbidity and mortality rate as the main index for safety evaluation. According to the previous reports, the morbidity and mortality rate for OTG is about 20%. Setting α=0.05, β = 0.2, and the noninferiority margin δ = 10%, the sample size required for each group is 90 cases. Considering that the maximum dropout rate for this clinical study is about 10%, the sample size is determined as 100 cases in each group.

### Study population

The patient inclusion and exclusion criteria are as follows:

#### Inclusion criteria

- Aged 18–75 years;
- Primary lesion is pathologically diagnosed as gastric adenocarcinoma, such as papillary adenocarcinoma, tubular adenocarcinoma, mucinous adenocarcinoma, poorly cohesive carcinoma (including signet ring cell carcinoma and other variants), and mixed adenocarcinoma;
- Clinical stage IA (T1N0M0) or IB (T1N1M0, T2N0M0) (According to AJCC-7th TNM staging system);
- Tumor located in the upper or middle third of the stomach, and curative resection is expected to be achievable by total gastrectomy with D1+/D2–10 lymphadenectomy (also apply to multiple primary cancers);
- No invasion to Z-line;
- BMI (Body Mass Index) < 30 kg/m^2^;
- No history of upper abdominal surgery (except for laparoscopic cholecystectomy);
- No prior treatment of chemotherapy, radiotherapy, targeted therapy, immunotherapy;
- No enlargement of splenic hilar lymph nodes;
- Preoperative performance status (ECOG, Eastern Cooperative Oncology Group) of 0 or 1;
- Preoperative ASA (American Society of Anesthesiologists) scoring: I-III;
- Sufficient organ functions;
- Written informed consent.

#### Exclusion criteria

- Preoperative examinations indicate that the stage of the disease is clinical stage II/III/IV;
- Preoperative examination indicate enlargement of perigastric or retroperitoneal lymph nodes (min diameter ≥ 1.0 cm);
- Women during pregnancy or breast-feeding;
- Synchronous or metachronous (within 5 years) malignancies;
- Body temperature ≥ 38 °C before surgery or infectious disease with a systemic therapy indicated;
- Severe mental disease;
- Severe respiratory disease;
- Severe hepatic and renal dysfunction;
- Unstable angina pectoris or history of myocardial infarction within 6 months;
- History of cerebral infarction or cerebral hemorrhage within 6 months;
- Continuous systemic steroid therapy within 1 month (except for topical use);
- Gastric cancer complications (bleeding, perforation, obstruction) that requiring emergency surgery;
- Patients are participating or have participated in another clinical trial (within 6 months).

### Randomized grouping

In this study, the central dynamic, stratified randomization method is adopted, and the factors including age, gender, BMI, and investigators, are considered. After each case is enrolled, the research center will arrange the research assistant to send the information of included cases (age, gender, and BMI) to the data center. After analyzing the case information by the center randomization department, the case grouping will be determined.

### Operation procedure

#### Laparoscopic total gastrectomy

The laparoscopic total gastrectomy is to be carried out with endotracheal intubation under general anesthesia. The peritoneal cytology examination should be first taken after entering the abdominal cavity. After the peritoneal cytology examination, the surgeon should explore the abdominal cavity to determine if there is any regional invasion or distant metastasis, including hepatic, peritoneal, mesenteric, or pelvic metastasis. Then, total gastrectomy with D1+/D2–10 lymphadenectomy will be performed according to the Japanese Gastric Cancer Treatment Guideline (Fourth edition, May. 2014) (Table 1). If clinical stage II or more diseases are diagnosed intraoperatively, D2 radical gastrectomy should be performed, and these patients will be excluded from this study. A proximal margin of at least 2 cm is recommended for T1 tumors, 3 cm is recommended for T2 or deeper tumors with an expansive growth pattern (Bormann I and II), and 5 cm is recommended for those with infiltrative growth pattern (Bormann III and IV). It is advisable to examine the proximal resection margin by Pathological Frozen Section routinely to confirm the negative proximal resection margin during the operation. The distal margin located in the duodenal bulb. The digestive tract reconstruction methods should be determined by the doctor in charge according to their experience and the specific intraoperative circumstances. Roux-en-Y reconstruction is recommended, and the linear and circle staplers are both acceptable. The laparoscopic operations inside abdominal cavity, including perigastric devascularization, lymph node dissection, and blood vessel ligation, must be performed using laparoscopic instruments. While, gastrectomy and digestive tract reconstruction could be completed with open approach through auxiliary incision (≤10 cm).

**Table 1:** D1+/D2–10 lymphadenectomy

Lymph nodes	Non-Cardia GC		EGJA	
	cT1N0	cT1N1/cT2N0	cT1N0/cT1N1	cT2N0
No. 1	✔	✔	✔	✔
No. 2	✔	✔	✔	✔
No. 3	✔	✔	✔	✔
No. 4	✔	✔		
No. 5	✔	✔		
No. 6	✔	✔		
No. 7	✔	✔	✔	✔
No. 8a	✔	✔		✔
No. 9	✔	✔		✔
No. 10				
No. 11p	✔	✔		✔
No. 11d		✔		✔
No. 12a		✔		
No. 19				✔
No. 20				✔

*GC* Gastric cancer, *EGJA* Esophagogastric junctional adenocarcinoma

#### Open total gastrectomy

The open total gastrectomy is similar to that of laparoscopic surgery, with the exception of operation performed under direct view.

### Postoperative care

The surgeons and resident doctors will evaluate the patients twice a day for whether there are events that affect the patients’ recovery course. The time to first ambulation and flatus, etc. will be recorded until discharged. Laboratory findings are recorded on the first, third, and fifth postoperative day.

Continuous intravenous postoperative analgesia is allowable but not mandatory within 72 h postoperatively. Its dose, type, and rate of infusion should be performed according to clinical routines and patient conditions. The repeated use of prophylactic analgesic is not permitted 72 h after surgery unless it must be used.

Postoperative fluid infusion (including glucose, insulin, electrolytes, vitamins etc.) or nutritional support (enteral/parenteral) is performed according to the clinical routines. After oral feeding, it should stop or gradually reduce fluid infusion/nutritional support.

A patient can be arranged for discharge when meet the following requirements: no postoperative complications, body temperature is less than 37 °C, the pain can be tolerated, and more than 1/3 normal diet can be oral intake.

### Quality control

To ensure the rationality of the surgical procedure, the quality of lymphadenectomy, the length of incision, and the integrity of specimen, a series of photographs of surgery (LTG and OTC) are taken for assessment. All photographs will be saved and submitted to CLASS data center within 1 week after the operation. The CLASS Research Committee will monitor and review regularly to ensure the quality of operation.

### Outcome measurements

#### Primary endpoint

The primary endpoint of the CLASS02–01 trial is noninferiority in the early operative morbidity and mortality, which are defined as the event observed within 30 days following surgery. The postoperative complications are defined and graded according to the grading system of Clavien-Dindo Classification. To measure this endpoint, the criteria are suggested in details as follows:

- Intraoperative complications
  1. Surgery-related complications: intraoperative hemorrhage (≥400 ml) and injury;
  2. Pneumoperitoneum-related complications: hypercapnia, mediastinal emphysema, subcutaneous emphysema, aeroembolism, and respiratory and circulatory instability caused by pneumoperitonum;
  3. Anesthesia-related complication.
- Early postoperative complications
  1. Surgery-related complications: wound complications (infection, effusion, dehiscence, poor healing), intra-abdominal active bleeding, digestive tract active bleeding, anastomotic stenosis, intestinal fistula, pancreatic fistula, chylous fistula, intra-abdominal abscess formation, gastroparesis, intestinal paralysis, intestinal obstruction, cholecystitis, pancreatitis, etc.
  2. System-related complications: pneumonia, pleural effusion, pulmonary embolism, cardio-cerebrovascular complications, deep venous thrombosis, urinary tract complications, catheter-related complications, condition of pain, etc.;
- Intraoperative or postoperative death

#### Secondary endpoint

The secondary endpoint of this study is the postoperative recovery course, which is assessed by time to first ambulation, flatus, liquid diet, soft diet, etc. In addition, the length of postoperative hospital stay will be recorded.

### Follow-up

The patients in both groups will be followed up 30 days after operation. A physical examination, a complete blood count, blood biochemical examination (albumin, prealbumin, total bilirubin, direct bilirubin, AST, ALT, creatinine, blood urea nitrogen, and blood glucose), and serum tumor markers (AFP, CEA, CA19–9, CA12–5, and CA72–4) analyses will be performed. In addition, imageological examination (total abdominal and pelvic enhanced CT, chest X-ray, upper gastrointestinal tract iodine imaging, gastroscopy, ultrasonography, whole body bone scan, PET-CT, etc.) will also be performed if needed. All the results will be recorded and evaluated by the specialist.

### Statistical analysis

Statistical analysis will be performed with SAS Software (version 9.3; SAS Institute, Cary, NC). The data are presented as the mean ± standard deviation for continuous variables and as a number for categorical variables. The statistical significance will be evaluated using χ^2^ test, Fisher’s exact test, t test, or rank sum test. The Newcombe’s method will be used to compare the 95% CIs (confidence intervals) for between-group differences of intraoperative and postoperative morbidity and mortality. All *P* values were two sided, and differences were considered significant at values of *P* < 0.05.

## Discussion

Incidence of adenocarcinoma at the upper third of the stomach has increased over the past two decades in Western countries [14], and in Asian countries, such as Japan, Korea, and China, an increasing trend of proximal gastric cancer is also observed during the past years [15]. For gastric cancer located on the proximal side of the stomach, both proximal gastrectomy (PG) and total gastrectomy (TG) can be considered, and each has advantages and disadvantages [16]. PG could offer the functional benefits relative to TG, such as less dumping syndrome, improved postoperative nutrition, and decreased likelihood of anemia. While, severe esophageal reflux is a potential postoperative complication for PG, even in need of second operation and reconstruction of the digestive tract [16]. Till now, various reconstruction methods have been developed to prevent postoperative esophageal reflux following PG, such as double tract reconstruction and jejunal interposition, however, the anti-reflux effect is uncertain [17]. So in this clinical trial, to maintain the nutritional status and quality of life of the patients, we recommended TG and Roux-en-Y reconstruction of the digestive tract, which remains the easiest solution with satisfactory functional results [16]. In addition, the role of complete resection of No. 10 nodes has long been controversial and one retrospective study in China proved that complete clearance of No. 10 nodes is no need for proximal gastric cancer with early stage [18], so in this clinical trial, the resection of No. 10 nodes is not required.

This CLASS02–01 trial is the first prospective randomized two-arm controlled study to determine the safety of LTG compared with OTG. Close-out will occur July 2018, at which time data will be analyzed. The findings from this study will contribute to evidence-based information about the safety of LTG with lymphadenectomy for gastric cancer. After safety assessment of the CLASS02–01 trial, the following clinical trial (CLASS02–02) will compare the long-term survival outcomes between LTG and OTG. Through these clinical trials, we hope to show that LTG with lymphadenectomy for gastric cancer is safe and feasible.

## Abbreviations

AFP: Alpha fetal protein
ALT: Alanine aminotransferase
ASA: American society of anesthesiologists
AST: Aspartate aminotransferase
BMI: Body mass index
CA: Carbohydrate antigen
CEA: Carcinoembryonic antigen
CLASS: Chinese laparoscopic gastrointestinal surgery study group
CT: Computed tomography
DSMC: Data and safety monitoring committee
ECOG: Eastern cooperative oncology group
EGJA: Esophagogastric junctional adenocarcinoma
GC: Gastric cancer
JCOG: Japan clinical oncology group
JLSSG: Japanese laparoscopic surgery study group
KLASS: Korean laparoscopic gastrointestinal surgery study group
LADG: Laparoscopic-assisted distal gastrectomy
LTG: Laparoscopic total gastrectomy
OTG: Open total gastrectomy
PET-CT: Positron emission tomography-computed tomography
PG: Proximal gastrectomy
RCT: Randomized controlled trial
TG: Total gastrectomy
